# Microbiome structure in a recirculating aquaculture system and its connection to infections in sturgeon fish

**DOI:** 10.14202/vetworld.2021.661-668

**Published:** 2021-03-18

**Authors:** Nurlan Khabibullovich Sergaliev, Murat Galikhanovich Kakishev, Nurbek Satkanuly Ginayatov, Farida Khamidullievna Nurzhanova, Evgeny Evgenievich Andronov

**Affiliations:** 1 Mahambet Utemisov West Kazakhstan State University, Uralsk, Kazakhstan, Nursultan Nazarbayev Avenue, 162, Uralsk, 090000, Republic of Kazakhstan; 2 Institute of Veterinary Medicine and Animal Husbandry, West Kazakhstan Agrarian-Technical University named Zhangir khan, Zhangir Khan Street, 51, Uralsk, 090009, Republic of Kazakhstan; 3 Laboratory of Microbiological Monitoring and Bioremediation of Soils, All-Russian Research Institute of Agricultural Microbiology, Podbelsky Highway, 3, Pushkin-8, Saint Petersburg, 196608, Russian Federation

**Keywords:** metagenomics, microbial contamination, microbiome, recirculating aquaculture system, sturgeon

## Abstract

**Aim:**

This study aimed to determine the bacterial composition at various stages of the temperature regime in a recirculating aquaculture system (RAS) to assess the pathological risk of a group of opportunistic pathogenic microflora.

**Materials and Methods:**

Water temperature, incidences of illnesses, and fish mortality were monitored, during the research period to identify the causes of pathogens in sturgeons. Analysis of the nucleotide sequences was performed using the quantitative insights into microbial ecology module. Sequence alignment in the analysis of the distribution of gene libraries was performed using the Unclust method. The RDP database was used for the taxonomic identification of operational taxonomic units.

**Results:**

The pattern of the contraction of infection among sturgeons bred in the RAS was established. A detailed analysis of the microbiome structure’s taxonomic features showed dominant taxa during the “artificial wintering” period and at a temperature optimum in industrial aquaculture. It was found that the main outbreaks of pseudomonosis occurred during this period in the RAS. With a decrease in temperature of the aquatic environment, the incidence of illness increased by 75% compared with the optimum temperature period. *Pseudomonas, Cetobacterium*, and *Lactococcus* were specific taxa characteristic for the “artificial wintering” period. *Xanthomonadaceae* and *Flavobacterium* were specific taxa characteristic for the optimum temperature.

**Conclusion:**

Consequently, the microbial structure was determined at different temperature regimes in a RAS, and the dominant communities were identified. The pattern of the contraction of infection caused by an opportunistic microflora (pseudomonosis) among sturgeons was established, allowing for the development and correction of treatment and preventive measures.

## Introduction

The technogenic method of growing sturgeon fish and their hybrids in a recirculating aquaculture system (RAS) is becoming popular globally. When the most optimal conditions for fish are created in the RAS, the recovery period after intravital caviar pumping is reduced, which increases the volume of production compared with natural caviar extraction. The development of this alternative method allows saving and restoring sturgeon stocks in natural reservoirs by reducing fishing pressure on their populations [1,2].

The aquarium complex is a multicomponent system that includes work units (breeding pools, mechanical and biological filtration, disinfection, and ozonation of recycled water), which pose a danger of potential pathogens accumulation. In industrial conditions, it is not always possible to control the level of microbial contamination. An increase in microbial concentration in the living environment leads to the emergence and spread of pathogens among sturgeons [3-7].

Microbial diversity at various stages of the filtration of recycled water in the RAS widely varies depending on many factors. The population density of cultivated aquatic organisms, water contamination with fish excreted material and food residues, water pH, and chemical parameters of water (the level of nitrates, nitrites, etc.) can affect the structure of the bacterial community [8,9]. Water temperature in the RAS affects the formation of the microbial composition. Proper temperature control optimizes the process of obtaining caviar [10].

It follows from the results of scientists that a significant proportion of diseases registered under RAS conditions is bacterial infections. There is a tendency in the form of a decrease in the number of diseases caused by obligate pathogenic microflora and, vice versa, a significant increase in the proportion of diseases caused by opportunistic bacteria (aeromonosis, pseudomonosis, myxobacteriosis, furunculosis, vibriosis, etc.) with a high mortality rate in 50-70% of fish; aquatic organisms that recover significantly lose weight and lag in development [11].

In connection with the specificity of forming microbial structure, depending on the above factors and an increase in the likelihood of the occurrence and spread of bacterioses among farmed sturgeons, the role of determining the microbiome structure in RAS increases. The study aimed to research the bacteriological composition at various stages of the temperature regime in the RAS system and link it to infectious disease outbreaks in sturgeon.

## Materials and Methods

### Ethical approval

The research protocol was discussed and approved at a meeting of the local ethical committee of the National Center for Biotechnology of the Science Committee of the Ministry of Education and Science of the Republic of Kazakhstan No. 4 dated February 10, 2020.

### Study period and location

The study was conducted from February to June 2020. The experimental production part of the study was conducted based on the aquarium complex of the Zhangir Khan West Kazakhstan Agrarian-Technical University.

### Study objects

We used RAS storage tanks intended for sturgeon breeding. The starting material was sampled from the floating loads of the biological filter (Sh), quartz filter (K), sediment from settling pool (O), breeding pool (B), and before and after final cleaning (F), taken at a system water temperature of 8°C during the artificial wintering and at an optimum temperature of 18°C. Broodstock and replacement livestock (4-5-year-old Siberian [*Acipenser baerii*], Russian [*Acipenser gueldenstaedtii*], and bastard [*Acipenser nudiventris*] sturgeons) grown in the RAS served as study objects.

### Study methodology

To achieve the aim, following tasks were determined:

- Monitoring of the water temperature in the RAS, monthly analysis of the incidence of illness, and mortality from infection of sturgeons
- Establishment of the level of diversity of microbial communities at different temperature regimes of water in the RAS
- Determination of diversity between microbial communities (beta-diversity) by cluster analysis.

To establish the patterns and seasonal dynamics of the contraction of infection, we studied the fish. The water temperature was monitored during “artificial wintering” to obtain caviar. Assessment of the intensity of the epizootic process was conducted by analyzing the incidence of illness and mortality among sturgeons in the studied period. The physiological condition was assessed by clinical fish examination, which considered the main indicators (changes in fish behavior, decreased appetite, and further cessation of nutrition), and expression of clinical features: Ulceration in various body parts of the body, inflammation of the anus, exophthalmia, etc. [12].

### Sampling procedure

To compile a collection of microbiome samples in the RAS system and analyze them more accurately, we took samples of circulating water in the RAS into individual sterile Falcon tubes (Eppendorf, Hamburg, Germany) in five replicates from two landing ponds No. 3 and 6, containing conditionally healthy sturgeons of the same sex and age categories. The selection of expert material was conducted by taking 200-300 mL of circulating water into a clean glass from various stages of the recirculation system and short-term settling. We drew 100 mL of water into a syringe to exclude large particles, screwed the cartridge, and pushed the water through the filter. We disassembled the cartridge, removed the filter, placed it on a clean filter paper, and then air-dried and stored it in a separate clean signed paper bag.

### Method for isolation of DNA from test samples

Bacterial DNA was isolated from samples according to the manufacturer’s instructions using an assay kit (MACHEREY-NAGEL NucleoSpin) from MACHEREY-NAGEL (Duren, Germany). For amplicon libraries preparation using PCR, amplification was performed for each sample with universal primers for the variable region of the 16S rRNA v3-v4 gene specific to several microorganisms, including bacteria and archaea (F515: GTGCCAGCMGCCGCGGTAA and R806: GGACTACVSGGGATAT).

### Molecular-biological research technique

PCR was performed in 15 μL of the reaction mixture containing 0.5-1.0 activity units of the Phusion Hot Start II High-Fidelity Polymerase and 1X Phusion buffer (Thermo Fisher Scientific, Waltham, Massachusetts, USA), 5 pcM of forward and reverse primers, 10 ng of DNA template, and 2 nM of each dNTP (Life Technologies, Carlsbad, California, USA). The mixture was denatured at 94°C for 1 min, after which 35 cycles were performed: 94°C for 30 s, 50°C for 30 s, and 72°C for 30 s. Final elongation was performed at 72°C for 3 min. PCR products were purified, according to the recommended Illumina method using AMPure XP (Beckman Coulter, Bray, California, USA). Further library preparation was performed, according to the MiSeq Reagent Kit preparation guide (Illumina, San Diego, California, USA) manufacturer’s instructions. Libraries were sequenced according to the manufacturer Illumina MiSeq instrument’s instructions using the MiSeq Reagent Kit v3 (600 cycles) assay kit with bidirectional reading (2 × 300 n).

###  Nucleotide sequence analysis

The taxonomic analysis of the nucleotide sequences of amplicon libraries was conducted using the quantitative insights into microbial ecology module (v.1.7.0).

During the analysis, libraries were allocated by identifiers, sequencing was verified, and nucleotide sequences were filtered. Sequences were combined into operational taxonomic units (OTU) using 97% similarity threshold, and nucleotide sequences were aligned using the Unclust method. The RDP database was used for the taxonomic identification of OTU [13].

## Results and Discussion

**Figure-1:**
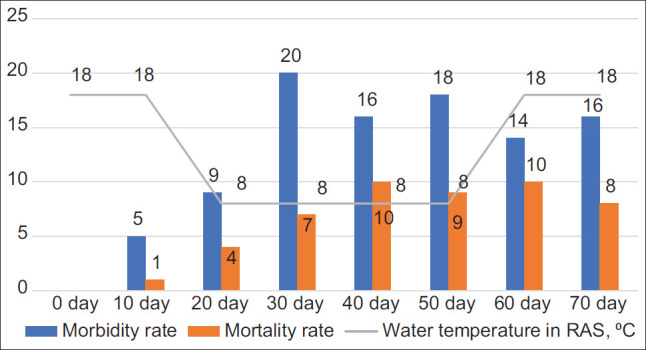
Effect of correction of seasonal biorhythms on the incidence of pseudomonosis in sturgeon fish bred in the recirculating aquaculture system.

During the study of patterns of the contraction of infection among sturgeons and considering the thermoregulation of water in the RAS, it was established that the main outbreaks of bacteriosis occurred during “artificial wintering” periods. During this phase, to optimize the endocrine system in the pre-spawning period, fish were kept in specially equipped pools with cooling systems (chillers). The water temperature was gradually reduced to 8°C at 1°C/day for 10 days. The fishes were kept for 30 days at 8°C, then slowly for 2 weeks, the temperature was raised to 18°C (Figure-1).

An analysis of the incidence of pseudomonosis showed an increase in this indicator with a decrease in the aquatic environment’s temperature by 75% compared with the optimum temperature period. After that, there was a decrease in the incidence rate associated with the sturgeons’ adaptation to the conditions of “artificial wintering,” but an increase in mortality to 50-60% from the moment of gradual decrease in the water temperature in the RAS and 90% from the beginning of the experiments conditioned by transiting the disease to a generalized form. The contraction of pseudomonosis in the RAS among sturgeons was also observed after increasing the temperature to 18°C, which can be explained by a decrease in the sturgeon’s general resistance after intravital caviar pumping.

**Figure-2:**
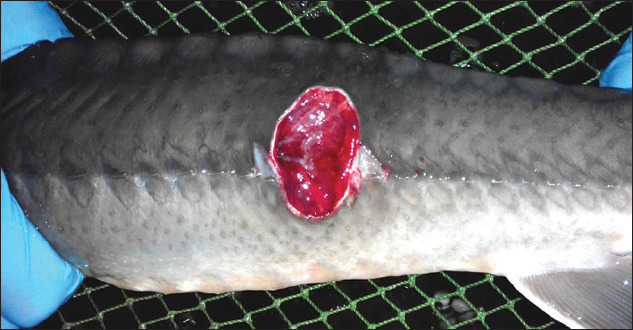
Ulcerative lesion in the dorsal part of the body of a sturgeon.

**Figure-3:**
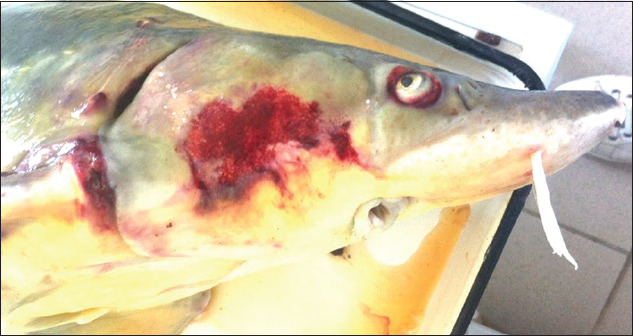
Lesion on the operculum and exophthalmia caused by pseudomonosis in a sturgeon.

The diagnosis of pseudomonosis was based on bacteriological studies, epizootological data, clinical features in fish in the form of ulcerative lesions on the surface of the skin (Figure-2), pathological changes, such as unilateral or bilateral exophthalmia (Figure-3), splenomegaly, multiple hemorrhages on the gastrointestinal tract, and biological test results on susceptible animals [14].

Consequently, outbreaks of pseudomonosis are directly associated with a decrease in water temperature in the RAS, when psychrophilic bacteria become active. The genus *Pseudomonas* is a prominent representative of this group, serving as the etiology of fish pseudomonosis [15].

**Figure-4:**
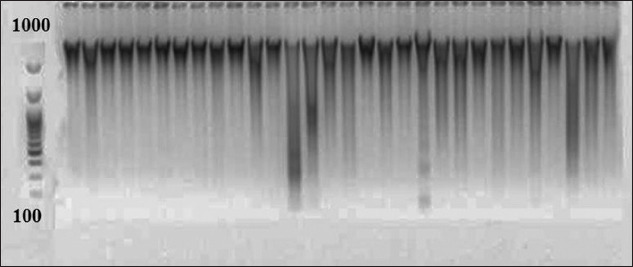
Electrophoresis of DNA isolated from samples taken from the recirculating aquaculture system.

The above results show that the water temperature in the RAS, which leads to changes in the structure of microflora, is considered a key factor complicating the epizootic situation and the complete elimination of the disease. Consequently, for a detailed study of the microbiome in RAS and revealing changes associated with temperature fluctuations, samples of the starting material were taken on the 35^th^ day from the beginning of the experiments – during the “artificial wintering” at 8°C (1) and on the 5^th^ day after temperature normalization to 18°C (2). After which, DNA was isolated from them. The quality of the extracted DNA was verified by electrophoresis (Figure-4). Then, the amplicons of the 16S rRNA gene were obtained.

**Figure-5:**
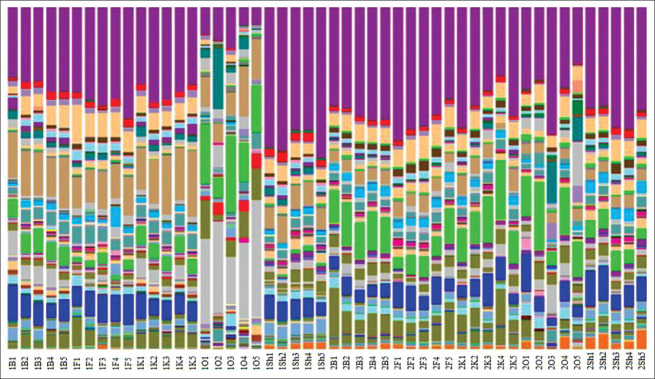
Taxonomic structure during the period of “artificial wintering” (1) and at a temperature optimum (2) in the recirculating aquaculture system.

The DNA yield was 50 μL with a concentration of 20-50 ng/μL, and the number of nucleotide sequences per library was 10-20,000. The number of detected prokaryotic taxa was 352 after the removal of minor components. In all cases, representatives of the phyla *Proteobacteria*, *Firmicutes*, *Bacteroidetes, Fusobacteria*, and *Actinobacteria* and a significant fraction of unclassified prokaryotes were dominant in the microbial community. Notably, representatives of the archebacterial phyla, *Thaumarchaeota*, were also identified in the microbial communities of reservoirs and purification lines. This particularly applied to the microbiota of sludge from the biofilters, where their proportion could be up to 5%. Figure-5 shows the total representation of the taxonomic structure of the microbiome during the optimum temperature and “artificial wintering” periods in the RAS.

An analysis of β-diversity was conducted using the *Bray-Curtis* similarity index to determine the similarity between communities. The analysis results showed several fundamentally essential phenomena: The most significant differences were found between different temperature regimes since there were clear differences during each stage of cleaning recycled water. For example, microbial communities from floating loads of biofilters had similar or even greater differences between the “artificial wintering” period and the optimum temperature periods than with other stages of water treatment. This fact shows the importance of microbiota analysis in industrial production since it significantly affects fish health and productivity.

There were differences in the structure of microbial communities between different water cleaning phases. High content of archaea was found in settling pools, which have the most isolated position, hence the most specific microbial community. Microbial communities of pools, quartz filters, and the final stage of water cleaning had significant similarities (although they had apparent differences) and differed only in quantitative indicators.

Table-1 shows the main indicators of the microbial diversity of communities (averaged over replicates). Microbial communities of the settling pools showed specific features of the diversity coefficients (lowest values for all coefficients).

**Table-1:** Coefficients of α-diversity of microbial communities.

Samples	Phylogenetic diversity		Species diversity		Universal coefficient		Evenness of distribution	
			
	8°C	18°C	8°C	18°C	8°C	18°C	8°C	18°C
Breeding pools (B)	80.90	72.97	824.75	808.45	7.66	7.48	0.99	0.98
Final cleaning (F)	80.01	86.65	748.17	984.78	7.78	7.90	0.99	0.99
Quartz filter (K)	83.52	62.60	901.16	659.13	7.64	7.28	0.99	0.98
Sediment from the settling pool (O)	32.69	43.14	298.45	416.11	5.18	6.46	0.91	0.96
Biological filter floating loads (Sh)	87.11	71.32	968.05	843.03	8.22	7.85	0.99	0.99

Pairwise comparisons of similar cleaning phases in different reservoirs were performed to analyze the reservoirs’ taxonomic features in more detail. The findings are described below.

**Figure-6:**
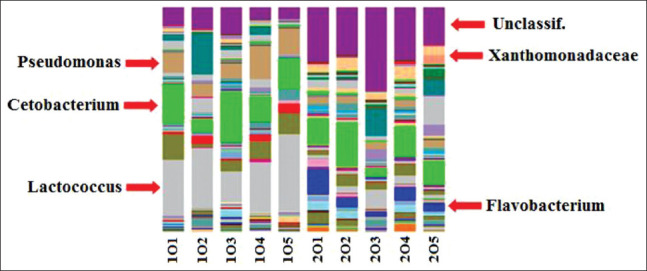
Comparative analysis of the microbial community of the settling pool during the “artificial wintering” period (1) and at the optimum temperature (2) in the recirculating aquaculture system.

**Figure-7:**
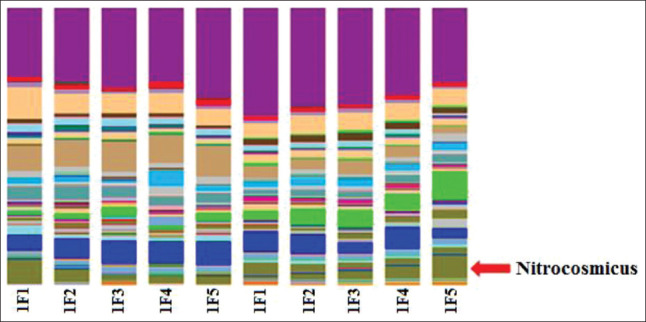
Comparative analysis of the microbial community at the final cleaning stage during the “artificial wintering” period (1) and at a temperature optimum (2) in the recirculating aquaculture system.

**Figure-8:**
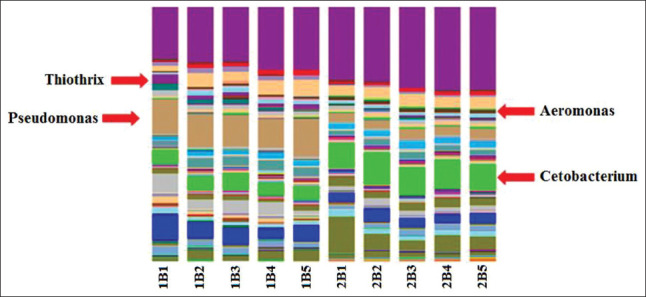
Comparative analysis of the microbial community during the “artificial wintering” period (1) and at a temperature optimum (2) in the recirculating aquaculture system.

1. *Pseudomonas*, *Cetobacterium*, and *Lactococcus* were specific taxa dominant in the breeding pools during the “artificial wintering” period; *Xanthomonadaceae*, *Flavobacterium*, and unclassified prokaryotes were specific taxa dominant in the breeding pools at the optimum temperature period (Figure-6)
2. There was a significant fraction of minor components, which were taxa specific for each filtration stage of recycled water
3. There were several differences between the communities (with the general similarity) after the final cleaning of recycled water in the reservoirs. For example, the proportion of the archebacterial taxon, *Nitrocosmicus*, was higher at a temperature of 18°C (Figure-7)
4. The difference, although to a lesser extent, persists after the final cleaning. This taxon belongs to the archaea that oxidizes ammonia, and a high content of this component in the studied pool was established during hydrochemical analysis (Table-2)
5. Comparison of the microbial communities of the recycled water showed a high degree of similarity with the only difference being the high titer of *Pseudomonas* and *Thiothrix* in the pool with water at a temperature of 8°C. *Aeromonas* and *Cetobacterium* were dominant in the pool with water at 18°C (Figure-8).

**Table-2:** The results of the monitoring of the levels of hydrochemical parameters in the RAS.

RAS sections	Mean value							

	“Artificial wintering” period				Temperature optimum			
	
	NH_4_	NO_2_	NO_3_	PO_4_	NH_4_	NO_2_	NO_3_	PO_4_
Breeding pools (B)	0.3	0.1	20	1	0.2	0.09	20	2
Final cleaning (F)	0.4	0.1	35	5	0.8	0.09	35	2
Quartz filter (K)	0.1	0.09	45	2	0.3	0.09	40	5
Sediment from the settling pool (O)	0.4	0.08	15	5	0.1	0.09	15	5
Biological filter floating loads (Sh)	0.2	0.06	15	2	0.1	0.09	35	2

RAS=Recirculating aquaculture system

Similar ratios were found in the microbiome structure in the sterilization state of recycled water.

A comparison of the initial and final stages of cleaning showed that the water’s taxonomic composition in pools changed during the cleaning process (the proportion of dominant prokaryotic taxa decreased during the cleaning process).

Such reservoir specificity was associated with the initial heterogeneity of microbial communities.

**Figure-9:**
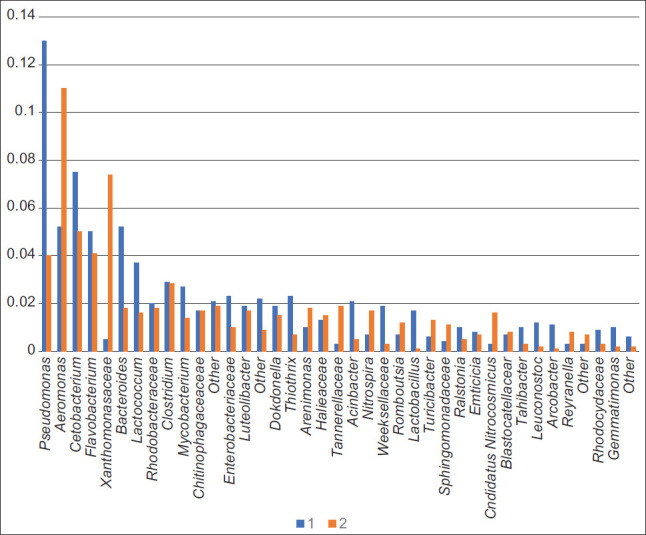
Comparison of microbiomes during the “artificial wintering” period (1) and at a temperature optimum (2) in the recirculating aquaculture system.

Figure-9 shows that the structure includes only those taxa that showed the maximum difference in different RAS temperature regimes. Among the taxa, the number of which varied significantly during different water temperature regimes in RAS, the genus *Pseudomonas* – the causative agent of sturgeon pseudomonosis is worth noting.

The causative agents of fish bacteriosis, in particular pseudomonosis, in contrast to the infectious origin of diseases of warm-blooded animals, have a pronounced feature due to adaptation to the biological properties of the fish organism, the body temperature of which directly depends on the temperature of the aquatic environment [16].

Thus, the optimum temperature for the reproduction of infectious agents and the manifestation of pathogenic properties in the fish organism has a rather wide fluctuation limit and serves as a complicating factor for the elimination of bacterial pathology among sturgeons in RAS. The pathogenic agents of bacterial diseases of warm-blooded animals are not adapted to such temperature changes [17].

Our studies and results of foreign studies allow us to believe that at a temperature optimum in the RAS, *Pseudomonas* was involved in nitrification and self-purification of water. However, their content in water increases to 5-6 times the initial value when the temperature in the system decreases (plus a period of “acclimatization” in sturgeons), which ultimately contributes to the outbreak and the massive spread of bacteriosis [18-20].

## Conclusion

The pattern of the contraction of infection caused by opportunistic microflora (pseudomonosis) was revealed among sturgeon fishes grown in the RAS. The main outbreaks of pseudomonosis occurred during “artificial wintering” when the temperature of the aquatic environment decreased to 8°C, and the bacteriosis etiology concentration exceeded the permissible microbial contamination levels.

Seasonal biorhythms effect on the incidence of pseudomonosis in sturgeon can be explained by the determination of the microbial structure results at various filtration stages of recycled water, and in the breeding pools, where the dominance of communities of *Pseudomonas*, *Proteobacteria*, *Firmicutes*, *Bacteroidetes*, *Fusobacteria*, *Actinobacteria*, and a significant fraction of unclassified prokaryotes was established. Archebacterial representative phylum, *Thaumarchaeota*, was also identified in the microbial communities of the pools and cleaning lines. The study of differences in the structures of microbial communities at different water cleaning phases revealed a specific microbial community typical for the settling pool – *Pseudomonas, Cetobacterium*, and *Lactococcus* – during the “artificial wintering” period, and unclassified prokaryotes, *Xanthomonadaceae* and *Flavobacterium* – at an optimum temperature in the RAS. Besides, the proportion of the archebacterial taxon, *Nitrocosmicus*, at the final cleaning stage at the optimum temperature was higher than at a low temperature in the RAS.

Results demonstrate several critical phenomena: Differences in the structure of microbiomes between the water cleaning phases in one reservoir and similar cleaning phases in different reservoirs.

Consequently, monitoring the natural microbiome’s state in the RAS using metagenomic analysis allows to determine the variation of the microbial composition in the system depending on the filtration types and temperature conditions and assessing the risk level of infections caused by a group of opportunistic pathogens, as well as, developing and adjusting treatment and preventive measures.

## Authors’ Contributions

NKS and MGK: Conception and design. NSG and FKN: Acquisition of data. NKS and EEA: Analysis and interpretation of data. NSG: Drafted the manuscript. NKS: Revised it critically. All authors read and approved the final manuscript.

## References

[1] Ginayatov N.S, Zalyalov I.N, Sergaliev N.K (2016). Vyyavlenie v uchastkakh ustanovki zamknutogo vodoobmena rezervuarov vozbuditelya infektsionnoi patologii osetrovykh ryb [Detection of reservoirs of the pathogen of the infectious pathology of sturgeon fish in recirculating aquaculture system]. Proceedings of the international scientific-practical conference of students, graduate students and young scientists. “Znaniya molodykh dlya razvitiya veterinarnoi meditsiny i agropromyshlennyi kompleksa strany. [Young knowledge for the development of veterinary medicine and the country's agro-industrial complex]”. Izdatel'stvo FGBOU VPO “SPbGAVM”, St. Petersburg.

[2] Ponomareva E.N, Sorokina M.N, Grigorev V.A (2014). Sostoyanie i Osobennosti Tovarnoi Akvakultury v Yuzhnom Makroregione Rossii [The State and Features of Commercial Aquaculture in the Southern Macroregion of Russia]. Proceedings of the International Scientific Conference, Aktualnye Voprosy Rybnogo Khozyaistva i Akvakultury Basseinov Yuzhnykh Morei Rossii. [Challenges in Fisheries and Aquaculture in the Basins of the Southern Seas of Russia]. Yuzhnyy nauchnyy tsentr RAN, Rostov-on-Don.

[3] Reinartz R, Bloesch J, Ring T, Stein H (2003). Sturgeons are more than caviar:A plea for the revival of sturgeons in the Danube River. Large Rivers.

[4] Arndt D, Xia J, Liu Y, Zhou Y, Guo A.C, Cruz J.A, Wishart D.S (2012). METAGEN assist:A comprehensive web server for comparative metagenomics. Nucleic Acids Res.

[5] Donkin M.J (1997). Bulking in aerobic biological systems treating dairy processing wastewaters. Int. J. Dairy Technol.

[6] Edgar R.C (2010). Search and clustering orders of magnitude faster than BLAST. Bioinformatics.

[7] Sergaliyev N.K, Kakishev M.G, Ginayatov N.S, Andronov E.E, Pinaev A.G (2019). The structure of the natural microbiome of sturgeon. Int. J. Eng. Adv. Technol.

[8] Hu L, Wang J, Wen X, Qian Y (2005). Study on performance characteristics of SBR under limited dissolved oxygen. Proc. Biochem.

[9] Ginayatov N.S, Zalyalov I.N, Absatirov G.G, Kakishev M.G, Zhunusov A.M (2017). Sravnitelnaya Otsenka Effektivnostei Metodov Obezzarazhivaniya Vody v Ustanovkakh Zamknutogo Vodosnabzheniya 232. [Comparative Evaluation of the Effectiveness of Water Treatment Methods in Closed Water Supply Systems]. Transactions of the N.E. Bauman Kazan State Academy of Veterinary Medicine.

[10] Golovanov V.K (2015). Temperatura i Zdorove ryb. Etiologicheskie Fiziologo-Biokhimicheskie i Immunologicheskie Aspekty. [Temperature and Fish Health. Etiological, Physiological, Biochemical and Immunological Aspects]. Proceedings of the 4th International Conference. “Problemy Patologii, Immunologii i Okhrany Zdorovya ryb i Drugikh Gidrobiontov. [Problems of Pathology, Immunology and Health Protection of Fish and Other Aquatic Organisms]. Borok, Moscow.

[11] Ozturk R.C, Altinok I (2014). Bacterial and viral fish diseases in Turkey. Turk. J. Fish Aquat. Sci.

[12] Bormotova S.V, Lartseva L.V, Rogatkina L.Y (1995). Sanitarnoe Sostoyanie Akvakultury Osetrovykh i Sredy Ikh Obitaniya [Sanitary Status of Sturgeon Aquaculture and their Habitat]. Fisheries. Series:Aquaculture. All-Russian Research and Design Institute of Economics, Information and Automated Fisheries Management Systems, Moscow.

[13] (2020). Ribosomal Database Project at Michigan State University.

[14] Sergaliyev N.H, Absatirov G.G, Tumenov A.N, Sariyev B.T, Ginayatov N.S (2017). Nosological description of fish pathologies in RAS. J. Pharm. Sci. Res.

[15] Brunetti R, Gasparri F, Marturano S, Prearo M (2006). *Pseudomonas fluorescens* infection in farmed Siberian sturgeon (*Acipencer baerii*). Ittiopatologia.

[16] Ture M, Alp H (2016). Identification of bacterial pathogens and determination of their antibacterial resistance profiles in some cultured fish in Turkey. J. Vet. Res.

[17] Capkin E, Terzi E, Altinok I (2015). Occurrence of antibiotic resistance genes in culturable bacteria isolated from Turkish trout farms and their local aquatic environment. Dis. Aquat. Org.

[18] Gulsen T, Akayli T, Korun J, Yardimci E (2010). A study on bacterial hemorrhagic septicemia in farmed young Russian sturgeon in Turkey (*Acipenser gueldenstaedtii*). J. Fish. Aquat. Sci.

[19] Ginayatov N.S, Zalyalov I.N, Absatirov G.G (2016). Identifikatsiya Vozbuditelya Infektsionnoi Patologii Osetrovykh Ryb v Usloviyakh Ustanovki Zamknutogo Vodosnabzheniya [Identification of the Pathogen of Infectious Pathology of Sturgeon Fish in Recirculating Aquaculture System]. Scientific Notes of Kazan State Academy of Veterinary Medicine named after N.E. Bauman. Vol. 226. Proceedings of the International Scientific Conference “Sovremennye Problemy Veterinarnoi i Agrarnoi Nauki i Obrazovaniya. [Modern Problems of Veterinary and Agricultural Science and Education] Dedicated to the 150^th^ Anniversary of the State Veterinary Service of Russia.

[20] Gridina T.S (2014). Osobennosti Mikroflory Biologicheskoi Sistemy Ustanovki Zamknutogo Vodoobespecheniya. [Features of the Microflora of the Biological System of Recirculating Aquaculture System]. Proceedings of the International Scientific Conference. “Aktualnye Voprosy Rybnogo Khozyaistva i Akvakultury Basseinov Yuzhnykh MOREI ROSSII. [Topical Issues of Fisheries and Aquaculture in the Basins of the Southern Seas of Russia]. Yuzhnyy Nauchnyy Tsentr Ran. Rostov-on-Don.

